# Simkania negevensis, an Example of the Diversity of the Antimicrobial Susceptibility Pattern among Chlamydiales

**DOI:** 10.1128/AAC.00638-17

**Published:** 2017-07-25

**Authors:** Manon Vouga, David Baud, Gilbert Greub

**Affiliations:** a Center for Research on Intracellular Bacteria, Institute of Microbiology, Faculty of Biology and Medicine, University of Lausanne, Lausanne, Switzerland; b Materno-fetal and Obstetrics Research Unit, Department Femme-mère-enfant, Maternity, University Hospital, Lausanne, Switzerland; c Infectious Diseases Service, University Hospital, Lausanne, Switzerland

**Keywords:** Chlamydiales, Simkaniaceae, intracellular bacteria

## Abstract

In past years, several Chlamydia-related bacteria have been discovered, including Simkania negevensis, the founding member of the Simkaniaceae family. We evaluated the antimicrobial susceptibility patterns of this emerging intracellular bacterium and highlighted significant differences, compared with related Chlamydiales members. S. negevensis was susceptible to macrolides, clindamycin, cyclines, rifampin, and quinolones. Importantly, unlike other Chlamydiales members, treatment with β-lactams and vancomycin did not induce the formation of aberrant bodies, leading to a completely resistant phenotype.

## TEXT

Rapid progress in diagnostic techniques has enabled the discovery of several novel Chlamydia-related bacteria, including Simkania negevensis. Mostly known for the pathogenic Chlamydia spp., the Chlamydiales order is now composed of at least 9 family-level lineages (1), each with specific biological characteristics. S. negevensis is the founding member of the Simkaniaceae family and represents an emerging pathogen previously associated with respiratory diseases, at least in the Middle East (2, 3). Infections were empirically treated with a macrolide-based regimen (4). Several differences regarding antimicrobial susceptibility have been highlighted among the different Chlamydiales family-level lineages (5, 6). Therefore, we investigated the antibiotic susceptibility of the Simkaniaceae family, which remains poorly studied, using S. negevensis as a model. We provide subsequent information on the evolution of antimicrobial resistance in this order, as well as potential therapeutic options.

Simkania negevensis strain Z was grown at 37°C in Vero cells in 25-cm^2^ cell culture flasks (Corning, USA), in Dulbecco's modified essential medium (DMEM) (PAN Biotech, Aidenbach, Germany) supplemented with 10% fetal calf serum (FCS), with 5% CO_2_. A 6- or 7-day-old coculture, diluted 1:1,000, was used to inoculate fresh A549 cells or Vero cells that had been seeded previously at 1.5 × 10^5^ cells/ml on a 24-well plate (Corning), as described previously (7). At 2 h postinfection, the medium was changed for medium containing 2-fold serial dilutions of various antibiotics. Antibiotic-free wells served as growth controls, while uninfected cells served as negative controls. Twelve antibiotics from 8 different classes were used in this study. MICs were defined as the minimal concentrations that prevented bacterial growth at day 6, compared to a control infection performed in the absence of antibiotics. Growth at day 2 was also assessed for β-lactams, fosfomycin, and vancomycin, to ensure the absence of effects due to instability of the compounds after 48 h at 37°C. An in-house specific quantitative PCR targeting the 16S rRNA gene was used to quantify S. negevensis DNA, as described previously (7). The absence of antibiotic toxicity toward cells was determined by examining the microplates using an inverted microscope (Zeiss Axiovert 25; Carl Zeiss). When solvents other than distilled water (i.e., dimethyl sulfoxide [DMSO], 0.1 M HCl, and 1 M NaOH) were used to suspend antibiotic solutions, the absence of effects of these solvents on S. negevensis growth was assessed.

Like other Chlamydiales species, S. negevensis was susceptible to macrolides, clindamycin, cyclines, and rifampin (Table 1). Interestingly, S. negevensis was susceptible to quinolones; while Chlamydiaceae are sensitive, other Chlamydia-related bacteria, such as Waddlia chondrophila, Parachlamydia spp., and Estrella lausannensis, are resistant (5, 6, 8). Previous work suggested that S. negevensis was resistant to ciprofloxacin (9). In that study, MICs were determined in amoebae, as the minimal concentrations that prevented amoebal lysis. The observed results might have been due to the presence of an efflux pump in amoebae and decreasing quinolone bioavailability. Although several mutations in the *gyrA* and *parC* quinolone resistance-determining regions (QRDRs) were identified, they differed from those observed in resistant Chlamydia-related bacteria, which may explain the observed absence of resistance (6, 9).

**TABLE 1:** Antibiotic susceptibility of Simkania negevensis, compared to others Chlamydiales^*a*^

Drug	MIC (μg/ml)					
	Simkaniaceae, S. negevensis (this study)^*b*^	Parachlamydiaceae, Parachlamydia acanthamoebae (8)^*c*^	Waddliaceae, W. chondrophila (5, 11)^*b*^	Criblamydiaceae, E. lausannensis (6)^*b*^	Chlamydiaceae	
					C. trachomatis (10, 21–24)^*b*^	Chlamydia pneumoniae (11, 21)^*b*^
Cyclines						
Tetracycline	2	ND	ND	0.25	0.25–0.5	0.125–0.5
Doxycycline	0.5	2–4	0.25	0.25	0.03–0.25	0.02–0.5
Lincosamide						
Clindamycin	1	ND	2–4	ND	0.25–2	ND
Macrolides						
Erythromycin	ND	0.06	ND	ND	0.02–2	0.02–0.25
Clarithromycin	ND	<0.06	ND	ND	0.02–0.125	0.004–0.125
Azithromycin	<0.06	ND	0.25	2	0.6–2	0.02–0.5
β-Lactams						
Penicillin derivatives	>1,000	>32	>32	>32	0.25–2	5
Ceftriaxone	>1,000	>32	>32	>32	16–32	ND
Phosphonic acid derivative						
Fosfomycin	>1,000	ND	500	ND^*d*^	500–1,000	>1,000
Glycopeptide						
Vancomycin	>1,000	ND	ND	ND	1,000	1,000
Fluoroquinolones						
Ciprofloxacin	4	>16	>16	32	0.5–2	1–4
Ofloxacin	1	>16	>16	16	0.5–1	0.5–2
Levofloxacin	0.5	ND	ND	ND	0.12–0.5	0.25–1
Rifamycin						
Rifampin	<0.06	0.25–0.5	ND	ND	<0.125 to 1	<0.125

a Shown are the MICs of various antibiotics against members of the Chlamydiales orders (5, 6, 8, 10, 11, 21–24). This table was adapted from reference 8 with permission. ND, not done.

b Tested in mammalian cells.

c Tested in amoebae.

d Criblamydiaceae present the Cys115-to-Asp substitution in the active site of MurA, which is known to confer resistance to fosfomycin in Chlamydia spp.

**FIG 1:**
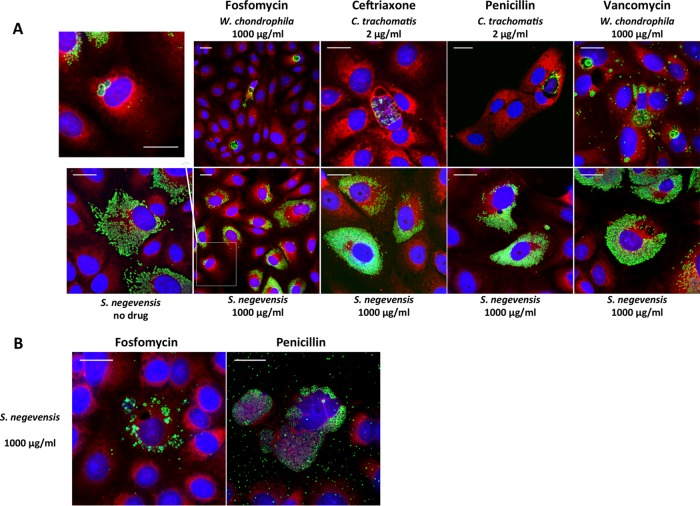
Effects of cell wall inhibitors on Simkania negevensis infection and morphology. The growth of S. negevensis was observed by immunofluorescence, in the presence or absence of cell wall inhibitors. (A) Effects of β-lactam, fosfomycin, and vancomycin treatment in Vero cells at 48 h postinfection. S. negevensis, Chlamydia trachomatis strain UW-3/Cx, and Waddlia chondrophila strain WSU 86-1044 (ATCC VR-1470) were detected using a polyclonal anti-S. negevensis rabbit antibody (1:2,500), a mouse anti-major outer membrane porin (MOMP) antibody (1:50) (ab20881; Abcam, Cambridge, UK), or an anti-W. chondrophila rabbit antibody (1:2,000), respectively (green), followed by a secondary antibody (Alexa Fluor 488-conjugated goat anti-mouse or anti-rabbit antibody [1:500]; Molecular Probes, Thermo Fisher Scientific, Waltham, MA), mammalian cells were stained with Texas red-conjugated concanavalin A (1:50) (red), and nucleic acids were stained with 4′,6-diamidino-2-phenylindole (DAPI) (1:1,000) (blue). (B) Effects of fosfomycin and penicillin treatment in Vero cells at day 6 postinfection.

S. negevensis was resistant (MICs of >32 μg/ml) to three kinds of cell wall inhibitors, i.e., β-lactams, fosfomycin, and vancomycin. Chlamydiales members lack the traditional peptidoglycan (PG) layer. However, partial susceptibility to β-lactams is observed among Chlamydia spp., which are known to form aberrant bodies when treated with penicillin derivatives (10), while W. chondrophila is susceptible to high doses of fosfomycin (11). Aberrant bodies represent enlarged forms of the bacterium, due to abnormal division despite persisting DNA replication (11). Therefore, we evaluated the morphology of S. negevensis particles treated with β-lactams, fosfomycin, and vancomycin, in immunofluorescence assays using an in-house rabbit polyclonal anti-S. negevensis antibody, as described previously (7). As shown in Fig. 1A, no abnormal morphological aspects of S. negevensis could be observed with β-lactam treatment, even with concentrations as high as 1,000 μg/ml. This contrasted strikingly with the abnormal morphology of Chlamydia trachomatis observed with 2 μg/ml β-lactams, making S. negevensis unique among Chlamydiales members. Indeed, W. chondrophila (in the Waddliaceae family) and E. lausannensis (in the Criblamydiaceae family) form aberrant bodies with β-lactam treatment (500 μg/ml) (6, 12). Furthermore, unlike W. chondrophila (11), S. negevensis replication was not inhibited by high doses of β-lactams (1,000 μg/ml) (Table 1). This difference could not be explained by the slower replicative cycle, as similar observations were made at day 6 postinfection (Fig. 1B). Several β-lactamase motifs are included in the S. negevensis genome (13) and may contribute to the phenotype. However, W. chondrophila exhibits partial sensitivity to high doses of β-lactams despite having a class C β-lactamase encoded in its genome (14).

Similarly to Chlamydia spp. (11), S. negevensis replication was not inhibited by high doses of fosfomycin, which targets the enzyme MurA (implicated in the early steps of PG biosynthesis). However, a small fraction of S. negevensis particles, which increased by day 6, showed abnormal morphological features consistent with aberrant bodies (Fig. 1A and B), although remaining significantly less important than observed for W. chondrophila (11). Chlamydia resistance to fosfomycin is suspected to be related to a single substitution (Cys115 to Asp) in the active site of MurA (11, 15). This mutation was not found in S. negevensis, supporting the observed partially sensitive phenotype. Finally, we did not observe aberrant bodies with vancomycin treatment, a drug that inhibits transpeptidation through high-affinity binding to the d-alanine precursor (Fig. 1A).

Recently, several works have demonstrated the presence of a modified version of PG, which is required for cell division (12, 16, 17), in Chlamydiales members, thus explaining their partial sensitivity to cell wall inhibitors. Interestingly, a recent study failed to isolate PG-like structures in S. negevensis (18), while such structures were identified in Protochlamydia amoebophila (18) and C. trachomatis (17). In the same work, incorporation of fluorescently labeled d-alanine could not be highlighted in S. negevensis (18), which correlates with the absence of vancomycin effects observed here. However, a previous work showed that, similarly to C. trachomatis, S. negevensis was susceptible to d-cycloserine, a molecule that inhibits the alanine racemase Alr and the alanine ligase Ddl, which are required for d-alanine formation (19). While a predicted Ddl enzyme is encoded in the S. negevensis genome, no Alr coding sequence is present, similarly to Chlamydiaceae (12). It is not known whether the serine hydroxymethyltransferase GlyA encoded in the S. negevensis genome could compensate for the absence of Alr, as described for Chlamydiaceae (20).

Despite the absence of PG-like structures, the activity of two PG-remodeling enzymes, NlpD and AmiA, was documented in S. negevensis (16), and enzymes implicated in PG biosynthesis are highly conserved among Chlamydiales members, including S. negevensis, which supports their crucial role (12). However, the different responses to different cell wall inhibitors, each targeting a specific step of PG biosynthesis, indicate that, despite the likely requirement for a modified form of PG for cell division, some significant differences exist in the PG biosynthesis pathway of S. negevensis, which might bring further insights into the mechanisms of Chlamydiales cell division.

In conclusion, in this work we highlighted several differences in the antimicrobial responses of S. negevensis, compared to other Chlamydiales members. Although the pathogenic role of Simkania spp. remains to be better defined, the precise knowledge of their antimicrobial susceptibility patterns provides significant information regarding the biology and evolution of the Chlamydiales order.
